# Differentiation of soft tissue and bone sarcomas from benign lesions utilizing ^18^F-FDG PET/CT-derived parameters

**DOI:** 10.1186/s12880-020-00486-z

**Published:** 2020-07-25

**Authors:** Bo Chen, Hongbo Feng, Jinghui Xie, Chun Li, Yu Zhang, Shaowu Wang

**Affiliations:** 1 Department of Radiology, The Second Affiliated Hospital of Dalian Medical University, Shahekou district, Zhongshan road, NO.467, Dalian, Liaoning Province People’s Republic of China; 2 Department of Nuclear Medicine, The First Affiliated Hospital of Dalian Medical University, Xigang district, Zhongshan road, No.222, Dalian, China

**Keywords:** ^18^F-FDG PET/CT, SUVmax, Heterogeneity factor, Soft tissue sarcoma, Bone sarcoma, Diagnosis

## Abstract

**Background:**

Accurate differentiation between malignant and benign changes in soft tissue and bone lesions is essential for the prevention of unnecessary biopsies and surgical resection. Nevertheless, it remains a challenge and a standard diagnosis modality is urgently needed. The objective of this study was to evaluate the usefulness of ^18^F-fluorodeoxyglucose (^18^F-FDG) PET/CT-derived parameters to differentiate soft tissue sarcoma (STS) and bone sarcoma (BS) from benign lesions.

**Methods:**

Patients who had undergone pre-treatment ^18^F-FDG PET/CT imaging and subsequent pathological diagnoses to confirm malignant (STS and BS, *n* = 37) and benign (*n* = 33) soft tissue and bone lesions were retrospectively reviewed. The tumor size, PET and low-dose CT visual characteristics, maximum standardized uptake value (SUVmax), metabolic tumor volume (MTV), total lesion glycolysis (TLG), and heterogeneous factor (HF) of each lesion were measured. Univariate and multivariate logistic regression analyses were conducted to determine the significant risk factors to distinguish sarcoma from benign lesions. To establish a regression model based on independent risk factors, and the receiver operating characteristic curves (ROCs) of individual parameters and their combination were plotted and compared. Conventional imaging scans were re-analyzed, and the diagnostic performance compared with the regression model.

**Results:**

Univariate analysis results revealed that tumor size, SUVmax, MTV, TLG, and HF of ^18^F-FDG PET/CT imaging in the STS and BS group were all higher than in the benign lesions group (all *P* values were < 0.01). The differences in the visual characteristics between the two groups were also all statistically significant (*P* < 0.05). However, the multivariate regression model only included SUVmax and HF as independent risk factors, for which the odds ratios were 1.135 (95%CI: 1.026 ~ 1.256, *P* = 0.014) and 7.869 (95%CI: 2.119 ~ 29.230, *P* = 0.002), respectively. The regression model was constructed using the following expression: Logit (*P*) = − 2.461 + 0.127SUVmax + 2.063HF. The area under the ROC was 0.860, which was higher than SUVmax (0.744) and HF (0.790). The diagnostic performance of the regression model was superior to those of individual parameters and conventional imaging.

**Conclusion:**

The regression model including SUVmax and HF based on ^18^F-FDG PET/CT imaging may be useful for differentiating STS and BS from benign lesions.

## Background

Soft tissue sarcoma (STS) and bone sarcoma (BS) are a rare group of mesenchymal origin diseases, which account for approximately 1% of adult malignant tumors [1]. At present, computed tomography (CT) and magnetic resonance imaging (MRI) are the preferred imaging techniques for clinical evaluation of such tumors [2, 3]. However, there are more than 200 diverse subtypes of soft tissue and bone tumors. Moreover, many lesions exhibit nonspecific morphological appearances; therefore, the discrimination between malignant and benign tumors using conventional imaging modalities is challenging and often leads to misinterpretation [4, 5]. Accurate discrimination between malignant and benign soft tissue and bone tumors is essential for the prevention of unnecessary pathological biopsies and unplanned surgical resections.

^18^F-fluorodeoxyglucose (^18^F-FDG) positron emission tomography/computed tomography (PET/CT) is a molecular imaging technique widely utilized to noninvasively quantify the glycolytic metabolism of tumors in vivo. It is typically employed during clinical assessment for tumor detection, staging and efficacy evaluation as well as prognosis prediction [6–8]. Maximum standardized uptake (SUVmax), metabolic tumor volume (MTV), and total glycolysis volume (TLG) are commonly used semi-quantitative parameters. Nonetheless, in practice, employing just one of the aforementioned parameters does not lead to effective distinction between malignant and benign lesions and frequently results in misinterpretation. Thus, a standard diagnosis modality is urgently needed [9]. In recent years, a quantitative intratumoral glucose metabolic heterogeneity indicator, namely heterogeneity factor (HF), which can be obtained by calculating metabolic volume-threshold function, has attracted significant attention [10]. Although numerous studies show that HF is closely related to the therapeutic response and prognosis of malignant tumors [11–13], discrimination between malignant and benign soft tissue and bone tumors utilizing this parameter remains unexplored.

Thus, the objective of the present study was to perform univariate and multivariate analyses to evaluate the usefulness of multiple ^18^F-FDG PET/CT-derived parameters and establish a multifactorial regression model for accurately discriminating STS and BS from benign lesions.

## Methods

### Patients and data management

This study was approved by the institution’s ethics review board. We retrospectively reviewed consecutive patients with soft tissue and bone lesions, who had undergone pre-treatment ^18^F-FDG PET/CT imaging at the First Affiliated Hospital of Dalian Medical University from April 2012 to December 2019. The pathological diagnoses were established by biopsy (*n* = 19) or analysis of the surgical specimen (*n* = 51). Data obtained from conventional imaging modalities, i.e., dynamic contrast enhanced-MRI (DCE-MRI) or enhanced CT, were collected simultaneously for cases when the PET/CT and conventional scans were performed within 2 weeks of each other. Patients who had undergone neoadjuvant therapy prior to the PET/CT examination and patients with a tumor size of < 1 cm were excluded from the study (partial volume effect was obvious). A total of 70 patients were included in the study. Among them, 39 were female and 31 were male. The median age of the patients was 58.5 (55.3 ± 13.8) years. The subjects were divided into the malignant (STS and BS, *n* = 37) and benign (*n* = 33) groups based on the 2013 WHO classification of soft tissue and bone tumors [14]. The pathological subtypes are summarized in Table 1.

**Table 1:** Histologic Type of the Tumors

Malignant tumors	n = 37	Benign tumors	n = 33
Liposarcoma	4	Schwannoma	7
Myxofibrosarcoma	4	Fibroma	5
Synovial sarcoma	4	Inflammatory myofibroblastic tumor	2
Hemangiosarcoma	5	Giant cell tumor of tendon sheath	2
Leiomyosarcoma	1	Giant cell tumor of bone	2
Rhabdomyosarcoma	1	Soft tissue hemangioma	1
Undifferentiated sarcoma	4	PHAT^a^ of soft parts	1
Pleomorphic sarcoma	1	Kaposi hemangioendothelioma	1
Spindle cell sarcoma	7	Langerhans histiocytosis	1
Osteosarcoma	2	Eosinophilic granuloma	1
Chondrosarcoma	4	Others	10

^a^**for pleomorphic hyalinizing angiostatin tumor**

### Image acquisition

All PET/CT imaging was performed utilizing the Biograph True-Point PET/CT scanner (Siemens Medical Systems, Germany). After fasting for at least 6 h, the patients were injected with 5.55 MBq/kg ^18^F-FDG and remained in a lying position in a quiet room for approximately 60 min. The PET/CT scan was acquired from the skull base to the proximal thigh. If necessary, both upper limbs and/or lower limbs were included. The patients were told to breathe quietly. CT scanning was first performed with 120 kV tube voltage and 60–80 mA tube current (Care Dose). Subsequently, a 3-dimensional acquisition mode with 1.5–2 min per bed position was adopted for PET imaging. The PET image data sets were reconstructed by subset expectation maximization using the CT image for attenuation correction.

### Image processing

**Fig. 1:**
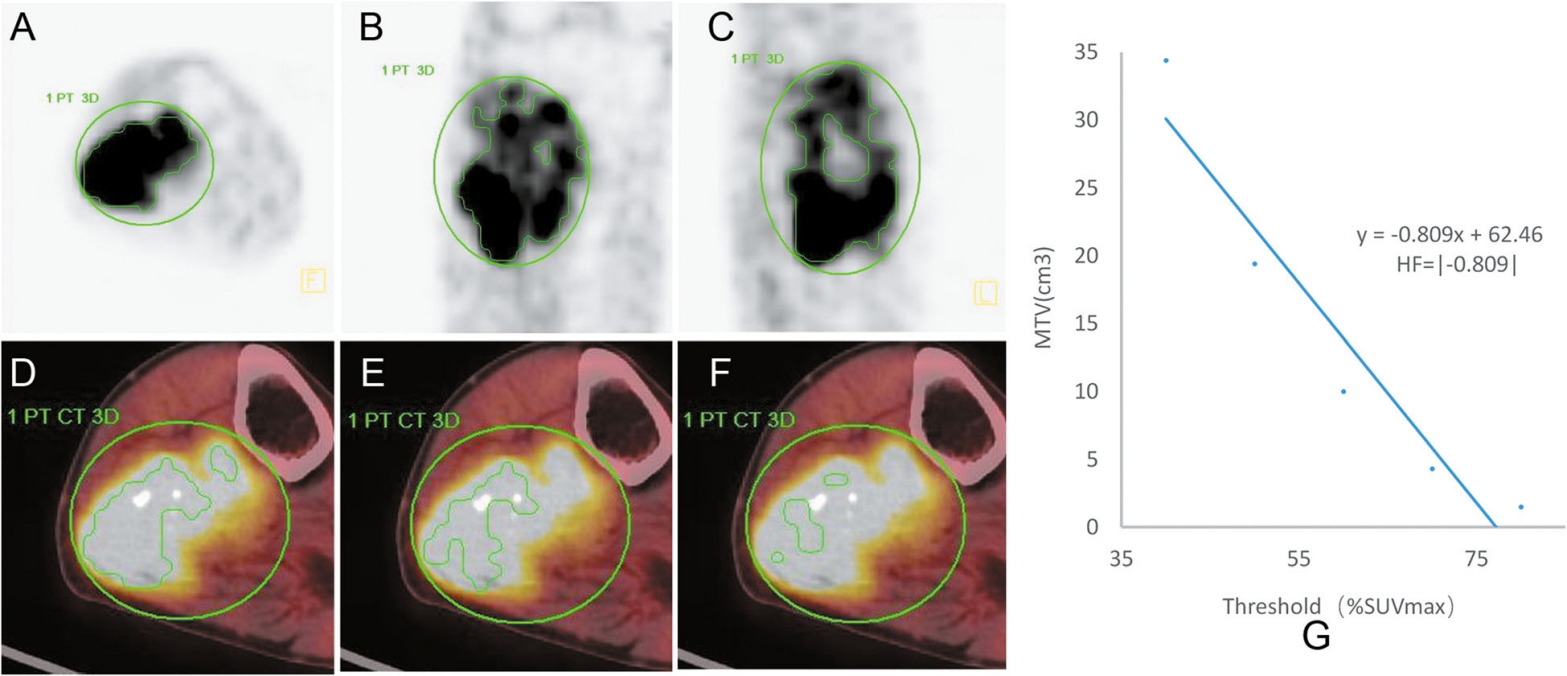
PET images depicting manually drawn VOI in three planes and the method used to calculate HF. (**a**) Axial plane. (**b**) Sagittal plane. (**c**) Coronal plane. (**d, e, f**) MTV decreasing gradually with increasing threshold (40, 50, 60% of SUVmax, respectively). (**g**) The slope of the threshold-volume function curve was calculated analogously to HF

The ^18^F-FDG PET/CT images were processed at a standard workstation (MMWP, Siemens) by two experienced nuclear medicine physicians. The measurement of the tumor size was performed by referring the PET/CT fusion image to confirm the tumor boundary. The largest plane (coronal, sagittal, or transverse) of the tumor was selected to measure its maximum diameter. Based on the PET and low dose CT scans, four visual characteristics of the lesions were obtained. These included ^18^F-FDG uptake, i.e., similar to the muscle tissue or significantly higher than the muscle tissue, ^18^F-FDG distribution, i.e., homogeneous or heterogeneous, lesion boundary, i.e., clear or obscure, and density, i.e., uniform or uneven. A semi-automatic method was utilized to delineate the tumor volume of interest (VOI) based on the threshold SUV. If necessary, tumor VOI was manually adjusted to cover the entire tumor tissue in three planes. Moreover, the normal tissue around the tumor and physiological uptake should be excluded as far as possible (Fig. 1). SUVmax was defined as the point of highest glucose metabolism within the VOI. MTV was expressed as the sum of voxel volumes of ≥40% SUVmax. TLG was calculated by multiplying MTV and SUVmean [15]. The derivative (dV/dT) of the metabolism volume-threshold function from 40 to 80% SUVmax was calculated from the linear regression curve [15]. Because of the derivative values were negative, the calculated derivative values were transferred to absolute values, which represented HF [10, 15]. The closer the derivative value was to the negative value, i.e., the greater the HF, the higher the heterogeneity of the tumor tissue. The conventional imaging scans were re-analyzed by two experienced radiologists.

### Statistical analyses

All statistical analyses were performed using SPSS 26.0 (IBM, Chicago, USA). Comparisons of continuous variables among groups were conducted via the Mann-Whitney U test. The chi-square test was utilized to analyze the intergroup differences of the categorical variables. Univariate and multivariable logistic regressions were adopted to identify independent predictors of malignant tumors. ROC curves were constructed and the areas under the curves (AUC) were established to evaluate the diagnostic value. The AUC values of individual parameters and their combination were compared employing the MedCalc software version 18.6.0. Based on the optimal cut-off values, the diagnostic accuracy was assessed by using sensitivity and specificity. Chi-square tests were utilized to compare percentages and *P* < 0.05 was considered statistically significant.

## Results

The univariate analysis results showed that the tumor size (malignant vs. benign: 7.5 ± 4.2 vs. 4.8 ± 5.2, *P* < 0.001), SUVmax (12.4 ± 9 vs. 7 ± 5.2, *P* < 0.001), MTV (57.7 ± 54.9 vs. 18.8 ± 16.5, *P* < 0.05), TLG (26.3 ± 513.8 vs. 81.1 ± 119.8, *P* < 0.001), and HF (1.39 ± 1.31 vs. 0.38 ± 0.35, *P* < 0.001) in the STS and BS group were all significantly higher than in the benign lesions group. Notably, the differences in the visual characteristics between the two groups were all statistically significant (*P* < 0.05). The majority of the malignant lesions were characterized by significantly higher FDG uptake, uneven FDG, unclear lesion boundaries, and uneven density, while the benign lesions exhibited contrasting features. Nonetheless, as shown in Table 2, there was no statistically significant difference in age (*P* = 0.911) and sex (*P* = 0.336).

**Table 2:** Comparisons of each Parameters between Malignant and Benign Groups

Characteristic	Grouping		Total	Statistical Magnitude	*P* value
	Malignant	Benign			
**Age(y)**	59 (55.8 ± 14.1)	56 (54.8 ± 13.6)	58.5 (55.3 ± 13.8)	*Z* = -0.112	0.911
**Sex**				*χ2* = 1.322	0.336
Male	23 (58.97%)	16 (41.03%)	39		
Female	16 (51.61%)	15 (48.39%)	31		
**FDG uptake**				*χ2* = 6.911	0.009
Higher	30 (81.1%)	17 (51.5%)	47		
Opposite	7 (18.9%)	16 (48.5%)	23		
**FDG distribution**				*χ2* = 8.036	0.005
Heterogeneously	28 (75.7%)	14 (42.4%)	42		
Homogeneously	9 (24.3%)	19 (57.6%)	28		
**Boundary**				*χ2* = 8.226	0.004
Obscure	30 (81.1%)	16 (48.5%)	46		
Clear	7 (18.9%)	17 (51.5%)	24		
**Density**				*χ2* = 6.708	0.010
Uneven	27 (73.0%)	14 (42.4%)	41		
Uniform	10 (27.0%)	19 (57.6%)	29		
**Size (cm)**	7 (7.5 ± 4.2)	3.8 (4.8 ± 5.2)	5.3 (6.2 ± 4.9)	*Z* = -3.490	< 0.001
**SUVmax**^a^	8.7 (12.4 ± 9)	4.7 (7 ± 5.2)	6.9 (9.8 ± 7.9)	*Z* = -3.507	< 0.001
**MTV**^b^**(cm**^**3**^**)**	36 (57.7 ± 54.9)	15 (18.8 ± 16.5)	24.8 (39.4 ± 45.6)	*Z* = -3.406	0.001
**TLG**^c^	251.4 (426.3 ± 513.8)	37.6 (81.1 ± 119.8)	102.4 (263.6 ± 417.7)	*Z* = -4.159	< 0.001
**HF**^d^	0.84 (1.39 ± 1.31)	0.54 (0.38 ± 0.35)	0.56 (0.92 ± 1.1)	*Z* = -4.171	< 0.001

^a^ for maximum standardized uptake value, ^b^ for metabolic tumor volume, ^c^ for total lesion glycolysis, ^d^ for heterogeneous factor, statistical description by n (%) or median (^*−*^*χ ± s*)

Only the variables with *P* < 0.05 determined from the univariable analysis were included in the logistic regression model. The variables included tumor size, visual characteristics, SUVmax, MTV, TLG, and HF. Based on multivariate logistic regression analysis, only SUVmax and HF were identified as independent risk factors for malignant tumors, and could be incorporated into the logistic regression predictive model. The odds ratios were 1.135 (95%CI: 1.026 ~ 1.256, *P* = 0.014) and 7.869 (95%CI: 2.119 ~ 29.230, *P* = 0.002), respectively (Table 3). Based on the above outcomes, the regression predictive model was constructed using the following expression: Logit (*P*) = − 2.461 + 0.127SUVmax + 2.063HF. The *P* values, i.e., the calculated probability, were generated from the regression model. The Hosmer-Lemeshow test indicated that the model fitted well (*χ2* = 7.025, *P* > 0 .05).

**Table 3:** Associated variables for discriminating STS and BS from benign lesions

Variables	Univariate			Multivariate		
	*β*	OR (95%CI)	*P*	*β*	OR (95%CI)	*P*
FDG uptake	1.395	4.034 (1.385–11.748)	0.011			
FDG distribution	1.440	4.222 (1.522–11.710)	0.006			
Boundary	1.561	4.554 (1.563–13.263)	0.005			
Density	1.299	3.664 (1.346–9.975)	0.011			
Size	0.159	1.173 (1.015–1.354)	0.030			
SUVmax^a^	0.127	1.135 (1.034–1.246)	0.008	0.127	1.135 (1.026–1.256)	0.014
MTV^b^	0.037	1.037 (1.012–1.063)	0.003			
TLG^c^	0.007	1.007 (1.002–1.011)	0.002			
HF^d^	2.115	8.288 (2.208–31.115)	0.002	2.063	7.869 (2.119–29.230)	0.002

^a^ for maximum standardized uptake value, ^b^ for metabolic tumor volume, ^c^ for total lesion glycolysis, ^d^ for heterogeneous factor, “OR” for odds radio, “CI” for confidence interval

**Fig. 2:**
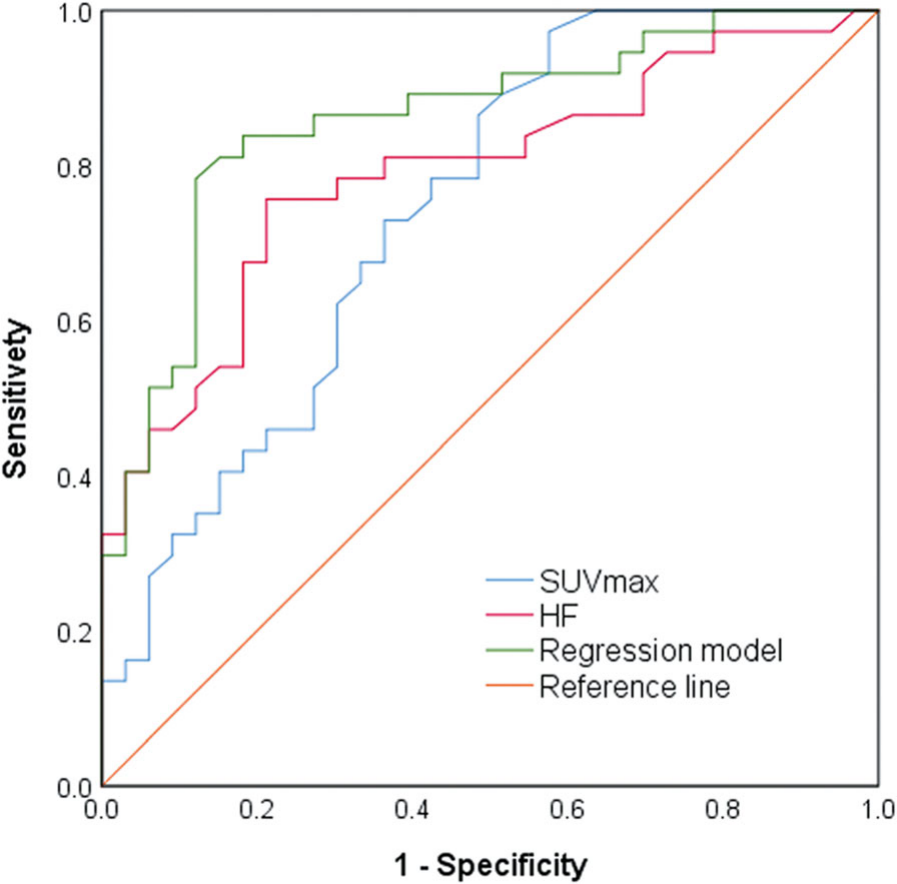
ROC curves of SUVmax, HF, and predictive regression model for differentiating malignant (STS and BS) and benign lesions. The AUC of SUVmax, HF, and the regression model were 0.744 (95%CI: 0.628 ~ 0.860, *P* < 0.001), 0.790 (95%CI: 0.684 ~ 0.896, *P* < 0.001), and 0.860 (95%CI: 0.771 ~ 0.948, *P* < 0.001), respectively

**Fig. 3:**
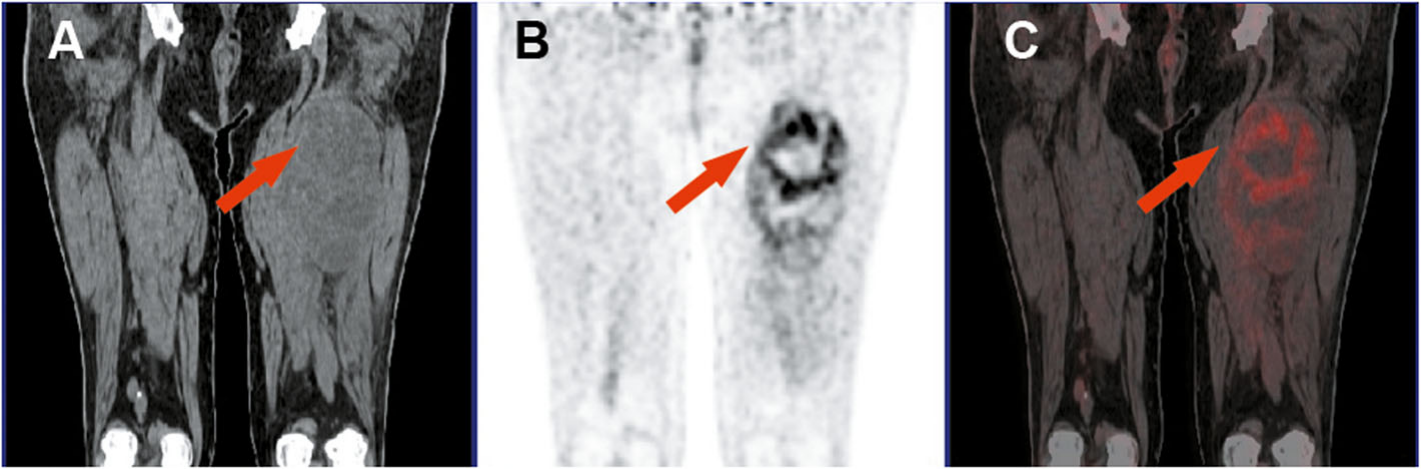
Liposarcoma of the left thigh. (**a**) CT-coronal plane. (**b**) PET-coronal plane. (**c**) PET/CT fusion image. As indicated by the red arrow, the density of the mass was equal or slightly lower than that of the adjacent muscle tissue. Moreover, the uptake of ^18^F-FDG increased significantly and heterogeneously. Tumor size = 16.9 cm, SUVmax = 8.3, HF = 2.93, TLG = 519.1, MTV = 123.6 cm^3^, and *P* value = 0.99

**Fig. 4:**
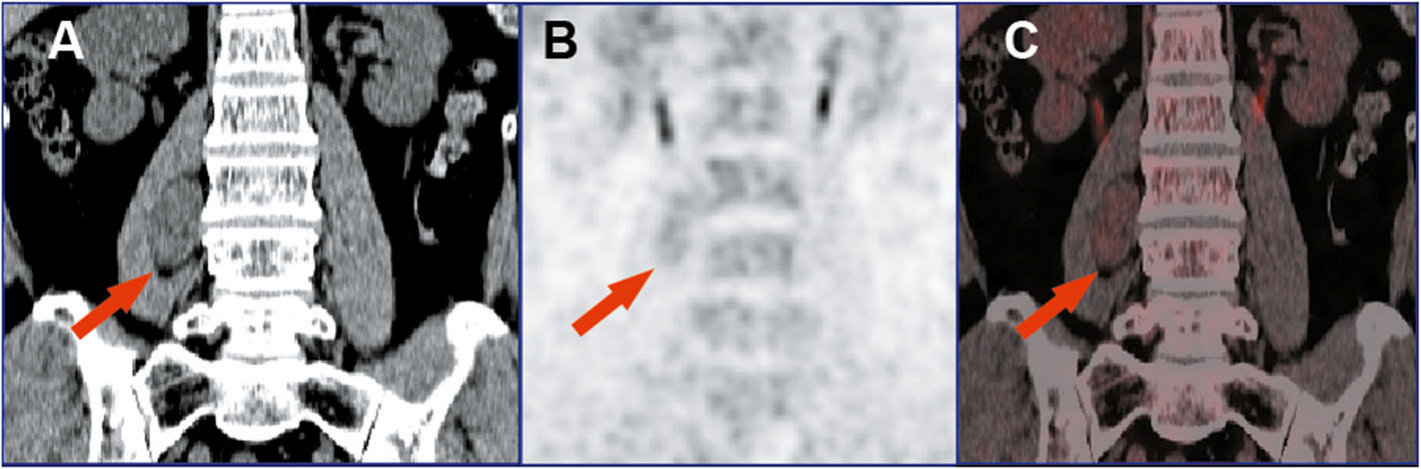
Schwannoma of the right psoas major. (**a**) CT- coronal plane. (**b**) PET- coronal plane. (**c**) PET/CT fusion image. As indicated by the red arrow, the density of the mass was slightly lower than that of the adjacent muscle tissue. Moreover, the uptake of ^18^F-FDG increased moderately and homogeneously. Tumor size = 2.5 cm, SUVmax = 3, HF = 0.36, TLG = 25.5, MTV = 15 cm^3^, and *P* value = 0.21

The ROC curves were plotted for the regression model *P* value, SUVmax, and HF to determine the effectiveness of the differential diagnosis (Fig. 2). The results revealed that the AUC of the model *P* values (AUC: 0.860, 95%CI: 0.771 ~ 0.948, *P* < 0.001) was higher in comparison to that of SUVmax (AUC: 0.744, 95%CI: 0.628 ~ 0.860, *P* < 0.001) and HF (AUC: 0.790, 95%CI: 0.684 ~ 0.896, *P* < 0.001). The difference between AUC of SUVmax and the model *P* values was statistically significant (*Z* = 2.277, *P* = 0.023). It was expected that the difference between the ROC curve of HF and the model would also be statistically significant. However, the difference between AUC of HF and the model *P* values was not significant (*Z* = 1.809, *P* = 0.070). Hence, the sensitivity and specificity were further compared statistically. The optimal cut-off values for model *P* value (0.47), SUVmax (5.95), and HF (0.46) were established. The sensitivity, specificity and accuracy were summarized respectively as following:

for model *P* value: 81.8, 83.8, 82.9%; for SUVmax: 69.7, 67.6, 68.6%; for HP: 72.7, 73.0, 72.9%. The results of the chi-square test demonstrated that the model *P* values exhibited higher specificity and sensitivity than SUVmax (*P* < 0.01) and the differences were statistically significant. Additionally, the specificity of the model *P* values was higher than in the case of HF (*P* < 0.01); however, the difference in the sensitivity was not statistically significant (*P* = 0.078). Compared to SUVmax and HF, nine false positive benign lesions and six false negative malignant lesions were correctly diagnosed by utilizing the regression model. Finally, the diagnostic performance of conventional imaging and the model *P* values were also compared. The outcomes demonstrated that the sensitivity was similar in both cases (19/24 vs. 20/24); however, the specificity of the model *P* values was significantly higher than that of conventional imaging (17/21 vs. 12/21). Representative cases were presented in Fig. 3 and Fig. 4.

## Discussion

^18^F–FDG PET/CT is widely used to characterize tumor glycolytic activity, which is a valuable marker of tumor biological behavior [16, 17]. In the present study, we assessed the usefulness of ^18^F–FDG PET/CT in differentiating STS and BS from benign lesions. Numerous studies have demonstrated that PET-derived semi-quantitative estimation parameters, such as SUVmax, MTV, and TLG, are valuable diagnostic indicators. Specifically, SUVmax is a marker of glucose metabolism of a single integrin in the tumor. On the other hand, MTV and TLG reflect the global metabolic activity of the tumor. However, the ability of individual parameters to discriminate between malignant and benign tumors in soft tissues and bones is not always adequate. Soft tissue and bone tumors are highly heterogeneous. Importantly, delays in the diagnosis have a negative impact on the final outcome [5]. Thus, the development of a simple and reliable imaging model to characterize biological behavior is critical to overcome the aforementioned limitations. In the present study, we comprehensively evaluated the feature parameters of ^18^F–FDG PET/CT imaging and constructed an effective model based on SUVmax and HF for differential diagnosis between malignant and benign soft tissue and bone tumors.

SUVmax, MTV, and TLG have been previously demonstrated as strong predictors of sarcoma cell proliferation and disease progression [17, 18]. Several studies have claimed that SUVmax and its retention index could both be used to differentiate between benign and malignant soft tissue or bone lesions [19, 20]. Nonetheless, SUVmax is not a precise indicator of the global metabolic activity of tumors. Moreover, a positive correlation between the FDG activity and the pathological grade of sarcoma has been established; however, the histological sub-types cannot be always distinguished accurately [21]. Specifically, some benign lesions may exhibit deceptively high FDG uptake, leading to indefinite diagnoses [22]. HF is another parameter obtained from PET images and was reported to reflect the intratumoral heterogeneity of ^18^F-FDG affinity. A study reported by Alipour R, et al. [23] showed that HF values for malignant parotid tumors were higher than for benign ones. Thus, HF was established as a reliable parameter for distinguishing between benign and malignant parotid tumors. Furthermore, Kim SJ, et al. [24] found that HF could be employed as a predictor for characterization of thyroid nodules. Nevertheless, the above studies only relied on univariate analysis, and multivariate analysis to eliminate the interaction among variables was not conducted. In the current work, the significant feature parameters between the malignant and benign lesion groups were screened according to the results of univariate analysis. The investigated parameters included tumor size, visual characteristics, SUVmax, MTV, TLG, and HF (all *P* values were < 0.05). Additionally, multivariate logistic regression analysis identified SUVmax and HF as the only independent risk factors for malignant tumors. Nakajo M, et al. [25] carried out a univariate analysis on 63 cases of musculoskeletal tumors using the cumulative SUV-volume histogram (CSH) method. The results showed that the AUC of CSH for malignant tumors was higher than that for benign ones, which was in agreement with the outcomes of the present study. However, this approach is analogous to the concept of dose-volume histograms for evaluating radiotherapy regimens, which uses PET/CT functional imaging data; thus, the clinical practicality is extremely limited. Xu R, et al. performed texture analysis for the differential diagnosis of bone and soft tissue lesions. The results obtained in this study revealed that utilizing optimal texture parameters combined with PET and CT imaging showed significantly better performance compared to SUVmax. Accordingly, the importance of combining parameters for differential diagnosis of diseases has been demonstrated [26]..

The vascular distribution and necrosis characteristics of each tumor cell population affect the growth rate [27]. The results obtained in the present study showed that the regression model AUC (AUC: 0.860, 95%CI: 0.771 ~ 0.948, *P* = 0.000) was higher than that of SUVmax (AUC: 0.744, 95%CI: 0.628 ~ 0.860, *P* = 0.000) and HF (AUC: 0.790, 95%CI: 0.684 ~ 0.896, *P* = 0.000). Based on the optimal cut-off values for the model *P* value (0.47), SUVmax (5.95), and HF (0.46), the diagnostic accuracy of individual parameters and their combination was assessed with respect to sensitivity and specificity. The outcomes demonstrated that the diagnostic performance of the regression prediction model combined with the SUVmax and HF parameters was considerably improved, particularly for specificity (all *P* values were < 0.01).

Generally, the ^18^F-FDG uptake is not homogeneous within tumors. The biological characteristics of tumors are determined not just by the tumor cells, but also by its microenvironment, including immune cells, endothelial cells, and tumor-related fibroblasts [28]. SUVmax reflects the highest glucose metabolism in tumor cells, while HF indicates the intratumoral heterogeneity of glucose metabolism. The combination of SUVmax and HF incorporates intertumoral structures, comprehensively reflecting the glucose metabolism inside the tumors and enabling more accurate characterization of the biological behavior. Previous research showed that the tumor size and volume are often considered as indicators of tumor malignancy [29, 30]. However, the multivariate logistic analysis conducted in this study demonstrated that when SUVmax and HF were simultaneously introduced to the regression model, the tumor size, MTV, and TLG were not statistically significant, indicating the presence of a certain overlaps and interactions between the parameters. The predictive value of tumor size and volume for a single location is limited. In addition, rapid proliferation of lesions indicates the presence of malignant tumors [31].

We subsequently compared the regression model with conventional imaging (DCE-MRI or enhanced CT). The results suggest that the sensitivity was similar for both approaches (20/24 vs.19/24, respectively). In contrast, the specificity for the model *P* values was significantly higher than for conventional imaging (17/21 vs.12/21, respectively). When the traditional images were reanalyzed, it was determined that two hematomas and a lesion rich in blood supply (Kaposi hemangioendothelioma and pleomorphic hyalinizing angiectatic tumor) were false positive. Notably, those were correctly diagnosed by the regression model. The present study is retrospective; therefore, most of the enrolled patients had suspected malignant lesions and underwent PET/CT imaging, the results of which may be subjective. The obtained results were sufficient to conclude that performing biopsy or surgical resection in patients with suspected malignant disease should be done with caution. Moreover, it was established that assessment using the PET/CT regression model prior to clinical decision might complement radiologic tomography, which is consistent with previous research [32].

Despite encouraging results, six false positive benign lesions (e.g.,giant cell tumors and inflammatory myofibroblastic tumors) as well as six false negative malignant lesions (e.g., myxofibrosarcomas) were determined by the regression model.^18^F-FDG is an analog of glucose and previous studies claimed that lesions with abundant infiltration of inflammatory cells or ones containing giant cells can display upregulation of hexokinase-2, leading to high FDG affinity [33]. Conversely, malignancies, which were rich in mucous matrix, usually exhibited insufficient glucose transporter expression and showed low FDG uptake [34]. In addition, a study by Lee AY, et al. showed that myxofibrosarcomas with a higher proportion of mucus are associated with a better prognosis [35]. Therefore, we speculate that this is the reason why the PET/CT imaging features of these tumors tend to be benign ones. Undeniably, biopsy remains as the gold standard for precise diagnosis.

The current study has certain limitations. Firstly, the sample size was not sufficiently large due to some STT pathological classifications being relatively rare. Secondly, the conducted study is a retrospective one, in which the pathological classification was confirmed by biopsy only in some of the cases. Moreover, the histological sub-type of several cases was not clearly defined. Nevertheless, we believe that the results of the present work provide a valuable reference for further research in this area. We propose a new concept, which effectively integrates the metabolic information obtained from ^18^F-FDG PET/CT imaging. The described approach can be used as a clinical standardized tool for the management of soft tissue and bone tumors. In particular, the methodology considerably enhances the specificity of imaging to avoid excessive pathological biopsies and unplanned surgical resections. A large sample of prospective cohort studies, involving more characteristic imaging parameters and histopathology factors should be carried out in the future.

## Conclusion

The regression prediction model established based on SUVmax and HF values obtained from ^18^F-FDG PET/CT imaging is a promising and noninvasive method, which was effectively utilized to distinguish soft tissue and bone sarcomas from benign lesions. The approach can be employed as an auxiliary diagnostic method to provide more reference information prior to treatment.

## Data Availability

The datasets used and/or analyzed of this study are available from authors on reasonable request.

## Abbreviations

STS: Soft tissue sarcoma
BS: Bone sarcoma
^18^F-FDG: ^18^F-fluorodeoxyglucose
PET/ CT: Positron emission tomography/computed tomography
SUVmax: Maximum standardized uptake value
MTV: Metabolic tumor volume
TLG: Total lesion glycolysis
HF: Heterogeneous factor
DCE-MRI: Dynamic enhanced magnetic resonance imaging
CT: Computed tomography
AUC: Area under receiver-operating characteristic curve
CSH: Cumulative SUV-volume histogram
